# Ranking occupations by their proximity to workers’ profiles

**DOI:** 10.1186/s41937-024-00125-2

**Published:** 2024-07-25

**Authors:** Mirjam Bächli, Hélène Benghalem, Doriana Tinello, Damaris Aschwanden, Sascha Zuber, Matthias Kliegel, Michele Pellizzari, Rafael Lalive

**Affiliations:** 1 Department of Economics, University of Lausanne, Lausanne, Switzerland; 2 Cognitive Aging Lab, Center for Interdisciplinary Study of Gerontology and Vulnerabilities, University of Geneva, Geneva, Switzerland; 3 Institute of Economics and Econometrics, University of Geneva, Geneva, Switzerland

**Keywords:** Worker profile, Occupational mismatch, Occupation recommendations, J21, J24, J62, J64

## Abstract

Information friction makes it difficult for job seekers to find new employment opportunities. We propose a method for providing individual-specific occupation recommendations by ranking occupations based on their proximity to the worker’s profile. We identify a set of twelve skills, abilities and work styles that capture the worker-oriented requirements of all occupations and discuss how to measure these items using online questions and tasks. We use the Euclidean distance between the measured items pertaining to a worker and the requirements of an occupation to measure the proximity between job seekers and occupations. We show that the proximity between job seekers’ profiles and their preunemployment occupation predicts their intention to change occupations, thus suggesting that our method captures a meaningful conceptualization of mismatch. We also show that our method generates recommendations that differ from the previous occupations of mismatched job seekers, thereby potentially expanding their search scope.

## Introduction

Friction is a ubiquitous feature of any labor market, and information regarding which jobs match a person’s profile is not readily available. To overcome such friction, job seekers often orient their job searches on their most recent job. However, this approach may not be a promising strategy due to mismatches pertaining to the previous job or because structural change has led to the disappearance of employment opportunities in the preunemployment occupation.

In recent years, job searches have increasingly shifted to the internet. Online job postings reduce the costs faced by employers and simplify communication with applicants. For job seekers, it can be challenging to process a large amount of information; however, the large pool of data available online also offers the opportunity for job seekers to search more effectively. Online job search platforms make it possible to incorporate user-specific advice in a cost-effective and scalable manner.

In this paper, we propose a tool that can be used to support job searches by providing individualized occupation recommendations, which rely on information regarding a worker’s profile. To measure the worker’s profile, we identify a small set of worker characteristics that capture the worker-oriented requirements of all occupations, and on that basis, we develop an online assessment tool to measure such characteristics. The resulting profile provides information concerning the worker that can be used to generate job search recommendations. We develop a Euclidean distance measure that allows us to rank occupations based on their proximity to the worker’s profile and to the occupation profile of the worker’s past occupation while accounting for work experience. The weight assigned to the worker’s profile and past work experience vary. The ranking of occupations can help job seekers understand the occupations that fit their profiles and can encourage them to focus on ensuring a low degree of mismatch between their profile and the occupation’s requirements in their job searches.

Our premise is that productivity depends strongly on the degree of match between workers and occupations. Workers differ in terms of characteristics that are not easily observable or measurable. Occupational requirements, on the other hand, can be described in taxonomies such as that provided by the Occupational Information Network (O*NET). To understand the notion of mismatch, we draw on O*NET’s comprehensive taxonomy of occupational descriptors, which provides us with the profiles of all available occupations. To measure workers’ profiles, we focus on a subset of descriptors that are important in most occupations but that exhibit sufficiently different degrees of importance to facilitate discrimination across different occupations.

Our approach can be summarized as follows. We first identify a set of occupational descriptors that are worker specific based on the O*NET taxonomy. We focus on abilities, skills and work styles because O*NET categorizes these characteristics as worker oriented. Within these three fields, we select descriptors that O*NET considers to be relatively important across all occupations and that simultaneously explain a relatively large share of the variation in importance values across occupations. The latter criterion helps us disregard descriptors whose importance is equally high across most occupations. Among the descriptors that satisfy these two criteria, we ultimately select twelve that can be measured within a reasonable time frame using an online assessment.

Second, we develop a self-administered online assessment to measure the twelve descriptors thus selected. The use of an online self-administered tool is cost-effective and can facilitate large-scale experiments. This assessment consists of a combination of established survey questions and cognitive tasks drawn from the field of psychology (Aschwanden et al., 2024). The resulting worker profiles can be compared to the occupation profiles associated with O*NET.

To our knowledge, we are the first to propose occupation recommendations based on measurements of individual profiles. A common alternative in the literature involves using the skills and abilities associated with workers’ previous occupations (Belot et al., 2019). However, past occupation(s) may not necessarily match a worker’s profile well (Manacorda & Petrongolo, 1999; Thisse & Zenou, 2000). From the perspective of job seekers who are forced to change careers, relying solely on the worker’s previous career path may not be desirable and may sometimes even be impossible.

Third, we propose a Euclidean distance measure that enables workers to identify occupations that match their profile well. This measure consists of two components. The first component is based on the twelve measured descriptors. It includes the distance between a worker’s profile and any given occupation profile as well as the distance between the profile of the worker’s previous occupation and any given occupation profile. The second component considers prior work experience more broadly and takes into account the fact that occupation mobility is limited, especially in the short term. It includes the distance between the worker’s previous occupation profile and any given occupation profile in terms of approximately 230 descriptors. The final measure of proximity that we use to generate recommendations is a weighted average of these two components, which feature weights that can be set by job seekers or policy makers, thereby offering a high degree of flexibility in the task of defining suitable occupations.^1^

We ultimately obtain a worker-specific distance for every possible occupation. Subsequently, we rank the occupations according to these distances and provide evidence regarding the properties of such rankings. We show that on average, 54% of the ten occupations that exhibit the shortest distances are within the same one-digit ISCO group as the reference occupation when focusing on the measured descriptors and disregarding the worker profile. This evidence indicates that our selection of descriptors is relevant.

We also discuss the relevance of the weights assigned to the measured profile and the previous occupation. We accomplish this goal by conducting a set of simulations that focus on one specific occupation, namely clerical office workers, which is the most common occupation in the Swiss labor market. We simulate a mismatch between the worker’s profile and any given occupation by creating fictional worker profiles and adding random noise to the occupation profile. We show that the probability of recommending a clerical office worker position rapidly decreases as the weight assigned to the worker’s profile increases. This evidence indicates that the provided occupation recommendations can facilitate the integration of new information in cases involving mismatch.

Our distance measure for occupation recommendations is inspired by the search and matching approach, with heterogeneity in terms of both jobs and workers with regard to their skills and informational friction (Miller, 1984; Şahin et al., 2014; Patterson et al., 2016; Guvenen et al., 2020; Lise & Postel-Vinay, 2020; Faberman et al., 2022). In this context, the match between workers and occupations is important, as mismatch reduces worker productivity. However, workers and occupations do not have all the information they need to identify productive matches. Our method intends to reveal a key information, namely whether a job seeker matches the requirements of an occupation.

Several approaches to the task of measuring transitions among occupations have been proposed in the literature. Gathmann and Schönberg (2010) suggest using the task distance among occupations to investigate occupation mobility. Belot et al. (2019), Schubert et al. (2020), Altmann et al. (2022) and Barbanchon et al. (2023) rely mainly on actual occupational transition data collected from surveys or resumes. As part of their ongoing work, Klaeui et al. (2024) construct an occupational similarity index based on the overlap in search requirements between two randomly chosen job advertisements. In contrast with these papers, we evaluate the profile of a worker in the context of an online assessment. Our worker-specific data capture characteristics that are likely to be transferable across occupations.

Recent intervention studies examine the question whether providing advice to job seekers improves their outcomes. Belot et al. (2019) offer participants access to a job search platform that aims to broaden job seekers’ spectrum of occupations. Dhia et al. (2022) and Briscese et al. (2022) evaluate online job search assistance tools offered by public employment offices in France and Australia, respectively, and focus on rich combinations of survey and administrative data regarding large samples of job seekers. Altmann et al. (2022) provide occupation advice online to job seekers in Denmark. Barbanchon et al. (2023) recommend job vacancies to job seekers using collaborative filtering algorithms. While we also provide online advice concerning where job seekers should apply, our occupation recommendations are based on a combination of worker-specific data and information regarding occupational requirements.

Naya et al. (2021) investigate the challenges associated with recommending jobs online. The paper discusses important targets for recommendation algorithms, such as the value of the new job, the probability of being offered the job or both. It also emphasizes the fact that congestion is an important element that recommendation algorithms must consider (Crépon et al., 2013). In our approach, the measured profiles of the job seekers are most likely to be different, resulting in heterogeneous recommendations. The proposed approach therefore leads to relatively little congestion compared to algorithms that make general occupation recommendations for a group of job seekers.

The remainder of this article is organized as follows. In Sect. 2, we develop a conceptual framework to describe the notion of occupational mismatch. In Sect. 3, we discuss the process of selecting the characteristics used to measure a worker’s profile. In Sect. 4, we present our distance measure for occupation recommendations, and in Sect. 5, we conclude.

## Conceptual framework

This section describes a simple framework for the effect of occupational mismatch on worker productivity.

We assume that jobs are organized into a set of occupations, which can be indexed by $o=1,...,O$. For the sake of simplicity, we further assume that jobs within occupations are identical, despite the evidence showing that skill requirements can vary substantially within occupations (Deming & Kahn, 2018). Employers offer a wage $p_{o}$ when the position is filled with a "perfect match", while a mismatch decreases that wage (Fredriksson et al., 2018). Specifically, a worker’s wage on the job is

$$y_{io}=p_o- d(i,o),$$

where *d*(*i*, *o*) is the mismatch between the profile of worker *i* and the requirements of occupation *o*.

We develop a method to measure the degree of mismatch between workers and occupations. Occupations are characterized by a set of descriptors, which can be indexed by $j=1,...,J$. A descriptor is a pair including a requirement $b_{oj}$ and its importance $w_{oj}$. Occupational descriptors are organized into (sub)domains, which can be indexed by $s=1,...,S$, including abilities, skills and work styles. Let $I^s_j = 1$ if descriptor *j* falls into subdomain *s* and $I_j^s=0$ otherwise. Workers *i* are characterized by their worker profile, which reflects the skill endowment by an individual to perform various tasks. The worker’s score with respect to descriptor *j* is captured by $c_{ij}$.

We capture the degree of mismatch between a worker and an occupation by focusing on the distance between the worker’s profile and the occupational requirements. First, we measure the degree of mismatch in subdomain *s* by using the weighted Euclidean distance.

$$d^s(i,o) = \sqrt{\sum _{j=1}^J (c_{ij}-b_{oj})^2 w_{oj} I^s_j}$$

The degree of mismatch in subdomain *s* is therefore large if the worker profiles deviate from the occupational requirements, i.e., $c_{ij}$ differs from $b_{oj}$, and if the requirement is important, i.e., $w_{oj}$ is high. Second, we capture the overall mismatch between a worker and an occupation by summing the distances across subdomains.

$$d(i,o) = \sum _{s=1}^S d^s(i,o)$$

The ideal approach is to measure proximity between worker and occupation profiles based on all available descriptors *d*(*i*, *o*). In real life, this approach poses a challenge since the descriptor space is very large and since only limited data regarding worker characteristics are available. We develop an approach that splits this large number of descriptors into a reduced set that we aim to measure using an online assessment as well as into a set that includes the remaining descriptors whose worker values are unknown but can nevertheless be proxied based on past occupation data.

We start by identifying a number of descriptors that (a) have a high value $w_{oj}$ across occupations and can therefore be viewed as important, (b) capture the variation in occupation requirements, and (c) are testable in an online format.^2^ Following this selection procedure, which is discussed in further detail in Sect. 3, we identify a reduced set of *M*-descriptors and assign them to a new subdomain *m*. Let $M_j = 1$ if the descriptor *j* is part of this set and $M_j=0$ otherwise. The distance between the worker and the occupation in the *M* subdomain is as follows:

1 $$\begin{aligned} d^m(i,o)=\sqrt{\sum _{j=1}^J (c_{ij}-b_{oj})^2 M_j} \end{aligned}$$

This *M*-distance captures a relevant component of the overall distance between worker *i* and any given occupation *o* by construction. Note that this distance is unweighted because all *M*-descriptors considered in this context are important. We then take the remaining descriptors and rewrite the subdomain distance $d^s(i,o)$ based on the set of descriptors *j* after removing the descriptors that are included in the *M* subdomain:

2 $$\begin{aligned} d^s_{-m}(i,o)=\sqrt{\sum _{j=1}^J (c_{ij}-b_{oj})^2 (1-M_j)I^s_j} \end{aligned}$$

By combining Eqs. (1) and (2), we can express the full distance between a worker and the occupation profile as follows:

3 $$\begin{aligned} d(i,o) \approx d^m(i,o) +\frac{w_L}{w^H} \sum _{s=1}^S d^s_{-m}(i,o) \end{aligned}$$

where $w^H$ is the average weight of the *M*-descriptors and $w^L$ is the average weight of the remaining descriptors. This approach shows that, in principle, the M-descriptor distance $d^m(i,o)$ can be used to approximate the full distance between a worker and an occupation *d*(*i*, *o*). Note that the ratio of the *M*-descriptor weights to the remaining weights captures the extent to which an approximation to *d*(*i*, *o*) using only the *M*-descriptors is accurate.

## Measuring worker profiles online

In this section, we first discuss the data used for the occupation profiles. We then present the criteria we use to select the twelve occupation descriptors that we measure in the online assessment for workers.

### Occupation descriptors

To understand occupational profiles, we draw on data from the Occupational Information Network (O*NET). O*NET characterizes occupations in a comprehensive way based on standardized surveys of incumbent workers, occupational experts, and trained analysts in the US economy.^3^ The occupation information is drawn from so-called descriptors, which provide information concerning different scales, such as those pertaining to “importance” and “level.” Level refers to the degree to which a given descriptor is needed in the context of an occupation, while importance refers to a more general conception of how important a descriptor is (even if the amount of the descriptor needed is small).^4^ The most recent data relate to 2019 and contain more than 450 descriptors pertaining to 923 occupations based on the 8-digit Standard Occupational Classification (SOC).

O*NET structures the occupation descriptors into six domains by topic. As shown in Table 1, these descriptors include three worker-oriented domains (worker characteristics, worker requirements and experience requirements) and three job-oriented domains (occupational requirements, workforce characteristics and occupation-specific information). The domains worker characteristics and occupational requirements are considered to be cross-occupational, while the domains experience requirements and occupation-specific information are considered to be occupation specific. In this paper, our goal is to measure a worker’s profile in a manner that is not tied to a specific occupation. We therefore focus on descriptors drawn from the worker-oriented domains that are related to individual traits: worker characteristics and worker requirements.

These domains are further divided into subdomains. We focus on descriptors in subdomains that allow us to link occupational profiles to workers’ characteristics. From the worker characteristics domain, we select abilities (“Enduring attributes of the individual that influence performance”) and work styles (“Personal characteristics that can affect how well someone performs a job”). We do not include occupational interests and work values because these descriptors are linked to preferences. From the worker requirements domain, we select skills, which is split further into basic skills (“Developed capacities that facilitate learning or the more rapid acquisition of knowledge”) and cross-functional skills (“Developed capacities that facilitate the performance of activities that occur across jobs”). While skills contain information regarding how individuals can work with certain types of knowledge and experience, the other two subdomains, i.e., knowledge and education, are likely to be (country-)specific and are not considered further.

**Table 1:** Structure of O*NET descriptors

Domain	Subdomains
*Worker-oriented domains*	
Worker characteristics	Abilities, occupational interests, work values, work styles
Worker requirements	Skills, knowledge, education
Experience requirements	Experience and training, skills – entry requirement, licensing
*Job-oriented domains*	
Occupational requirements	Work activities, organizational context, work context
Workforce characteristics	Labor market information, occupational outlook
Occupation-specific information	Title, description, alternate titles, tasks, technology skills and tools

This table summarizes the structure of the O*NET content model, including its domains and subdomains. A full description of O*NET’s content model is available at this link. Examples of the descriptors and specific occupations can be found at this link in the section “Browse by O*NET Data”. Source: O*NET

### Selection of measured descriptors

The most recent version of O*NET includes 52 abilities, 35 skills and 16 work styles. Our goal is to identify a subset of descriptors that can describe a worker’s profile sufficiently to facilitate discrimination across occupations. We consider the following criteria in our selection of items to measure: importance, information content and online testability.

**Importance.** All descriptors of the three subdomains abilities, skills and work styles provide information regarding their importance for a given occupation on a scale ranging from 1 (not important) to 5 (extremely important). To obtain a measure of importance, we compute the average importance of each descriptor across all occupations. To prompt the reader’s intuition in this context, the five descriptors that feature the overall highest importance are associated with the subdomain work styles and include attention to detail, dependability, integrity, cooperation and self-control (see Appendix Fig. 7).

**Information content.** We investigate the amount of the total variance in the importance value that each descriptor explains. Principal component analysis (PCA) allows us to identify a subset of descriptors that preserve as much information from the full set as possible. We focus on the eigenvector of the first principal component, at which most of the information is compressed. Specifically, this factor explains 40.41% of the total variation.^5^ To obtain a measure of informativeness, we consider the correlations between the components of the first principal component eigenvector and the descriptors. Among these components, higher absolute values (i.e., high loadings) indicate that the associated descriptor is more closely related to the variation compressed in the first principal component regarding the importance of a descriptor. We conduct PCAs with regard to all subdomains to avoid overlapping information. The five descriptors that exhibit the highest information content overall are written expression (ability), reading comprehension (skill), writing (skill), written comprehension (ability) and active listening (skill).

Descriptors that exhibit relatively high importance and explain a high share of the total variation are candidates for the assessment of the third criterion, i.e., “online testability.” In contrast, descriptors that exhibit relatively high importance in many occupations are disregarded because they do not allow us to discriminate among occupations. For example, the ability of selective attention has a mean importance of 3.05, with values ranging between 2.25 and 3.75. It is relatively important in many different occupations, including for chemical processing plant operators (ISCO 3133, importance value 3.75), security guards (ISCO 5414, importance value 3.67) and lawyers (ISCO 2611, importance value 3.50). Similarly, the ability of near vision exhibits a mean importance of 3.59, with values ranging between 2.38 and 4.12, and various occupations are associated with high requirements regarding this ability, including among pharmacists (ISCO 2262, importance value 4.12), aircraft pilots (ISCO 3153, importance value 4.04) and printers (ISCO 7322, importance value 4.00). We thus focus on a subset of descriptors that are important in many occupations while simultaneously exhibiting sufficient variation in their degree of importance.

**Online testability.** To develop a worker-specific measure of these occupation descriptors, we investigate whether the set of important and informative descriptors can be measured using a self-administered online assessment that can be completed within a reasonable amount of time. Further criteria for online testability include construct validity (i.e., the availability of a test/survey that accurately measures what it is supposed to measure) and reliability (i.e., consistency of a measure over time). We are aware that determining what is measurable entails a certain amount of arbitrariness. To address this issue, in Sect. 4, we discuss the correlations between occupation profiles based on the measured descriptors and those based on an extensive set of alternative descriptors.

**Selected descriptors.** We select twelve descriptors after applying the criteria of importance, information content and online testability. Table 2 provides an overview of this set of descriptors as well as a brief summary of the aspects that they cover in the final column. From the abilities subdomain, we draw the following five descriptors: fluency of ideas, memorization, inductive reasoning, category flexibility and perceptual speed. From the skills subdomain, we draw the following three descriptors: reading comprehension, time management and monitoring. Finally, from the work style subdomain, we draw the following four descriptors: adaptability, tolerance to stress, leadership and self-control. Figure 8 in Appendix shows the occupation profile of a “clerical office worker”, which is the most common occupation in Switzerland (ISCO 4110), in terms of the importance of the measured descriptors. Self-control, stress tolerance and adaptability are considered to be relatively important, whereas memorization, fluency of ideas and perceptual speed are viewed as less important.

**Fig. 1:**
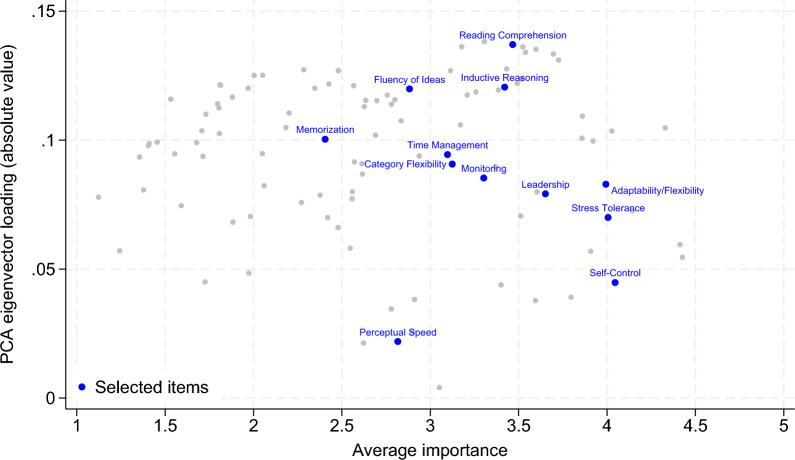
Importance and information content of the ability, skill and work style descriptors. *Note:* The x-axis shows the average importance of each descriptor across all occupations. The y-axis shows the loading of the first principal component eigenvector in absolute values. The descriptors selected to measure a worker’s profile are shown in blue. The gray dots represent all other descriptors from the subdomains of abilities, skills and work styles. Source: O*NET, own calculations

The choice of descriptors requires a certain amount of discretion. Naturally, the practical feasibility of the measurement of a descriptor and reliance on established tests are highly important when designing an assessment. Despite some degree of subjectivity, Fig. 1 shows that the selected descriptors are located in the upper right corner, thereby exhibiting a relatively high average importance (x-axis) and loading of the first principal component eigenvector (y-axis). The only exception to this rule is the descriptor of perceptual speed, which exhibits an average importance at the mean but a relatively low first principal component loading. However, perceptual speed exhibits a high loading of 17.7 in the second principal component.

**Table 2:** Selected measured descriptors

Descriptor	Subdomain	Content
Category flexibility	Ability	Generating or using different sets of rules for combining or grouping things in different ways.
Fluency of ideas	Ability	Ability to generate novel ideas concerning a topic. The number of ideas is important (as opposed to their quality, correctness or creativity)
Inductive reasoning	Ability	Combining pieces of information to produce general rules or conclusions (which includes identifying relationships among seemingly unrelated events).
Memorization	Ability	Remembering information such as words, numbers, pictures and procedures.
Perceptual speed	Ability	Ability to compare similarities and differences among sets of letters, numbers, objects, pictures, or patterns quickly and accurately. The items to be compared may be presented simultaneously or in succession.
Monitoring	Skill	Monitoring and assessing the performance of oneself, others, or organizations with the goal of making improvements or taking corrective action.
Reading comprehension	Skill	Understanding written sentences and paragraphs in work-related documents.
Time management	Skill	Managing one’s own time and the time of others.
Adaptability	Work style	Being open to change and to diversity in the workplace.
Leadership	Work style	Willingness to lead, take charge, and offer opinions and direction.
Self-control	Work style	Maintaining composure, keeping emotions in check, controlling anger and avoiding aggressive behavior, even in very difficult situations.
Tolerance to stress	Work style	Accepting criticism and dealing with high-stress situations calmly and effectively.

This table shows the twelve measured descriptors in the light of the criteria of importance, information content and online testability. Source: O*NET

### Online assessment of worker profiles

We develop an online assessment to measure a worker’s profile based on these twelve descriptors. The assessment can be completed within approximately one hour on a personal computer with an internet connection. No auxiliary means are needed. Finally, the assessment is self-administered and does not require any personal interactions between the research team and participants. These features ensure that the assessment is cost-effective and scalable.

The online assessment consists of a combination of survey questions and tasks. Survey questions can be more targeted, but responses may be biased if participants select options that sound good rather than those that correspond to them. On the other hand, task-based exercises are less manipulable but generally require more time to complete than survey questions. Moreover, these exercises can be irrelevant to the actual abilities and skills that are required in the work context. We use survey questions to measure less tangible capabilities pertaining to work styles. These descriptors refer to behavior in specific situations (e.g., high stress or difficult situations) and are too time-consuming to assess using tasks. To measure abilities and skills, we use a variety of task-based exercises because these capabilities are considered to be difficult to report through self–assessment from a worker’s perspective, as in the case of IQ tests. Various questions and tasks included in the online assessment are described in Aschwanden et al. (2024).

### Mismatch, occupational mobility intentions and age

The conceptual framework developed in Sect. 2 implies that job seekers who are employed in occupations that exhibit many differences from their profiles are likely to search for occupations that more closely resemble their profiles. Consider a job seeker *i* who worked in occupation $o_i$ prior to entering unemployment. This worker earned a wage $y_{io_i}=p_{o_i}-d(i,o_i)$. Suppose that this worker considers a new job in occupation *o* that offers a wage $y_{io} = p_o-d(i,o)$. The wage difference between the job offer and the previous job is $y_{io}-y_{io_i}=p_o-p_{o_i} +d(i,o_i)-d(i,o)$. Thus, we expect job seekers associated with a large mismatch $d(i,o_i)$ to obtain the most benefits from switching occupations.

We next discuss the extent to which the proposed distance measure $d(i,o_i)$ actually captures mismatch. We use data concerning the actual worker profiles of approximately 1,500 job seekers from the study conducted by Benghalem et al. (2023), in which context each participant completed an online assessment of the measured descriptors as discussed in Sect. 3.3.^6^ We then use administrative data regarding the participants’ preunemployment occupations and calculate the Euclidean distance $d^m(i,o_i)$ between the workers’ profiles and those of their previous occupations (see Eq. 1). The data also contain information concerning the (main) occupation *o* that the job seekers target in their job searches. This information was collected through mandatory and standardized interviews that included case workers associated with Public Employment Services and job seekers in the initial phase of their unemployment.

**Fig. 2:**
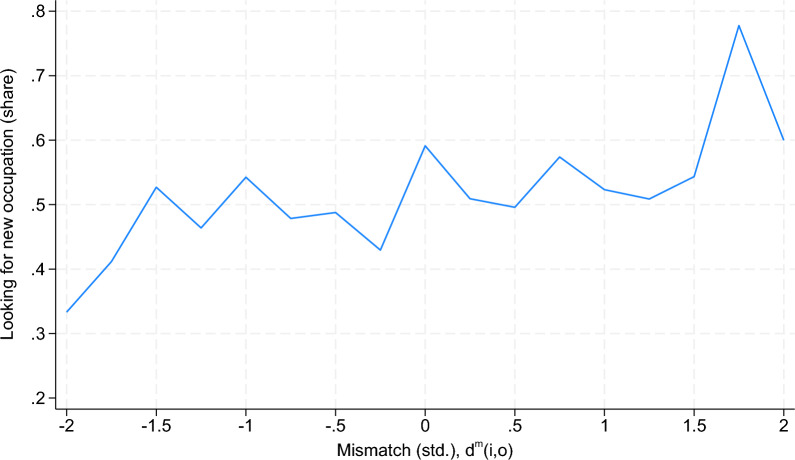
Mismatch and occupational mobility intentions. *Note:* This figure shows the (standardized) distance between the workers’ profiles and their previous occupation on the horizontal axis and the proportion of job seekers who are seeking positions in an occupation that differs from their previous occupation on the vertical axis. Source: Benghalem et al. (2023), own calculations

By comparing the previous occupation with the target occupation, we construct a measure of occupation switching intentions that takes a value of 1 if the worker’s target occupation differs from the preunemployment occupation and a value of 0 otherwise. Figure 2 shows the relationship between the distance measure $d^m(i,o_i)$ and the share of job seekers who would prefer to change occupations. The job seekers who exhibit the highest distance report a 60% likelihood of changing occupations, i.e., a much higher value than the 34% reported by the job seekers who exhibit the shortest distance. This evidence suggests that our notion of mismatch strongly predicts the intention to change occupations.

**Fig. 3:**
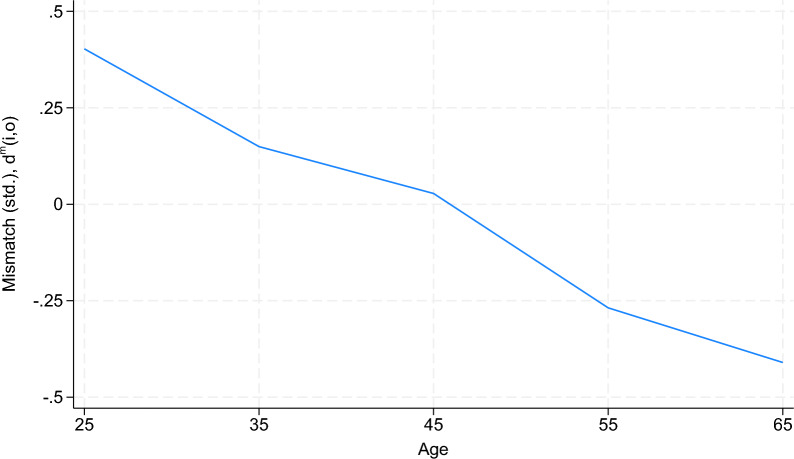
Mismatch and age. *Note:* This figure shows age groups on the horizontal axis. The intercept 25 refers to workers who are younger than 30 years old, the intercept 35 to workers between the ages of 30 and 39,..., and the intercept 65 to workers who are 60 years old or older. The mean (standardized) distance between the workers’ profiles and their previous occupations is shown on the vertical axis. Source: Benghalem et al. (2023), own calculations

To improve our understanding of the groups of job seekers that are most likely to experience mismatch, we calculate the average mismatch of job seekers by age group. Figure 3 shows that job seekers who are younger than 30 years exhibit a standardized level of mismatch that amounts to 0.4 of a standard deviation. Job seekers between the ages of 30 and 39 years, as indicated by the midpoint of 35, exhibit an average mismatch that amounts to approximately 0.2 of a standard deviation. Mismatch decreases nearly linearly with age, reaching a level of $-$0.4 among job seekers who are 60 years old or older. This evidence suggests that workers tend to exhibit the highest degree of mismatch early in their working lives and that match quality improves through occupational mobility as workers age. This finding is consistent with the stylized facts presented in Topel and Ward (1992).

## Worker-specific occupation recommendations

In this section, we present the distance measure used to derive the ranking of occupations based on their proximity to workers’ profiles. We then discuss the measure’s components and the role that they play in the process of identifying the occupations that are most closely related to a worker’s profile.

### Distance measure

Following the conceptual framework shown in Sect. 2, we develop a measure that allows us to compute the proximity between a worker’s profile and any given occupation in a comprehensive and flexible way.

As shown in Eq. 4, our worker-specific distance measure *d*(*i*, *o*) consists of two components that account for different sets of worker characteristics. The first component is based on the twelve measured descriptors *m*, while the second component is based on a number of other descriptors *s*:

4 $$\begin{aligned} d(i,o)= & {} \frac{[\alpha \times d^m(i,o) + (1-\alpha ) \times d^m(o_i,o)] + \beta \times \sum _{s=1}^{S}d^s_{-m}(o_i,o)}{1+S\beta }\nonumber \\= & {} \frac{d^m(o_i,o) + \alpha \times [d^m(i,o) - d^m(o_i,o)] + \beta \times \sum _{s=1}^{S}d^s_{-m}(o_i,o)}{1+S\beta }, \end{aligned}$$

where *i* is an individual, *o* is any occupation, $o_i$ is the previous occupation and $0 \le \alpha , \beta \le 1$.

The first component consists of the Euclidean distance between a worker’s profile and the profile of any occupation $d^m(i,o)$, as weighted by $\alpha$, as well as the Euclidean distance between the profile of the worker’s previous occupation and any occupation $d^m(o_i,o)$, as weighted by $(1-\alpha )$.^7^ The second component $\sum _{s=1}^{S}d^s_{-m}(o_i,o)$ is the sum of the Euclidean distances between the previous occupation profile and any occupation. This component reflects general work experience in terms of 232 occupation descriptors across nine subdomains *s*.^8^ Finally, we take the average of the weighted distance matrices to obtain *d*(*i*, *o*).^9^ Occupations that exhibit a shorter distance *d*(*i*, *o*) are more closely related to a worker’s profile.

**Fig. 4:**
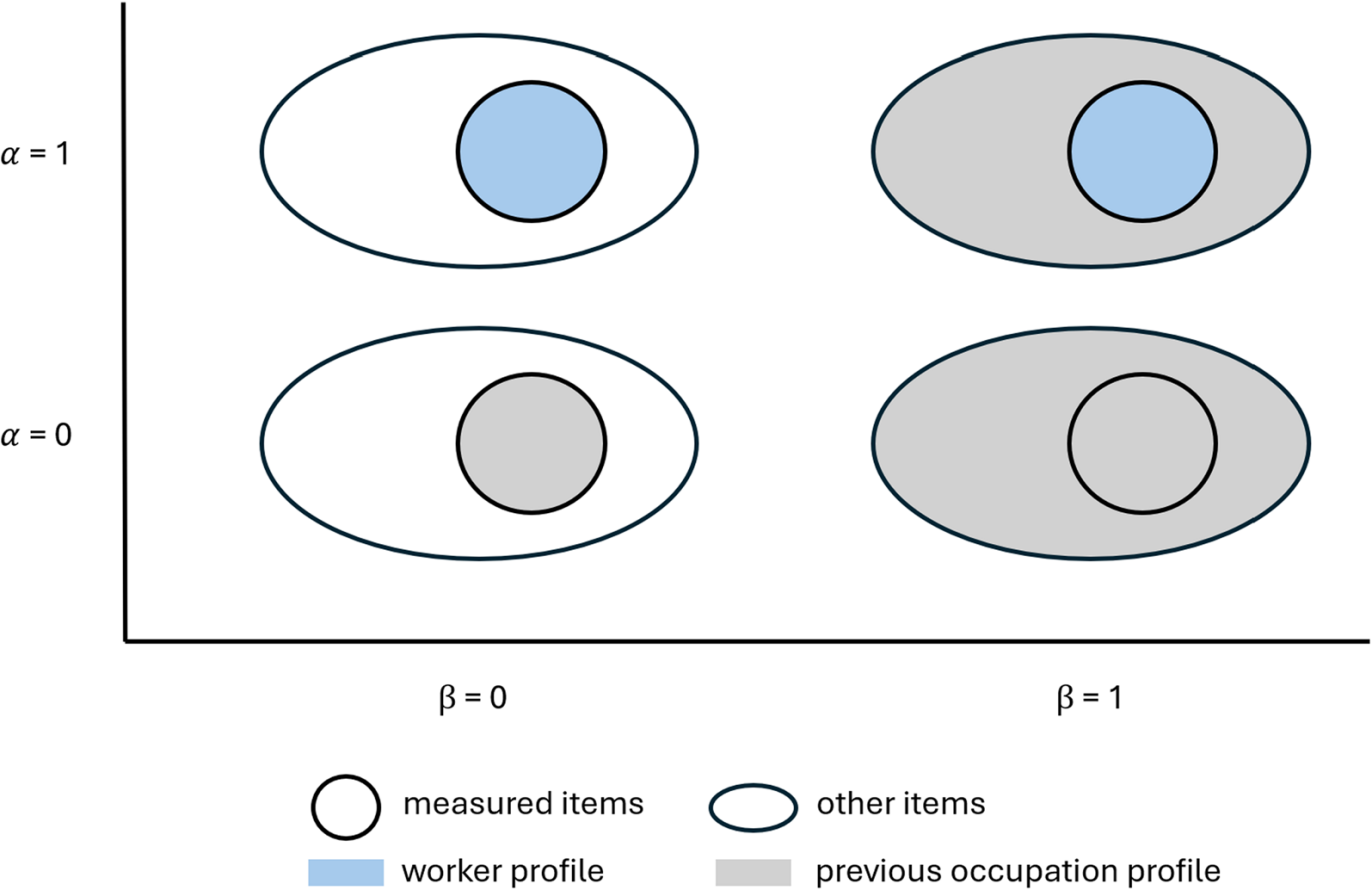
Data used for the distance measure depending on parameters $\alpha$ and $\beta$. *Note:* This figure shows the data that are included in the distance measure depending on the parameter specifications of $\alpha$ and $\beta$. The small circle in blue corresponds to $d^m(i,o)$ in Eq. 4. The small circle in gray corresponds to $d^m(o_i,o)$. The large oval circle in gray corresponds to $\sum _{s=1}^{S}d^s_{-m}(o_i,o)$

Figure 4 provides an overview of the information components depending on the parameters $\alpha$ and $\beta$. The parameter $\alpha$ specifies the weight on the worker’s profile versus the previous occupation profile in terms of the measured descriptors. The parameter $\beta$ indicates how far away the occupation search area may be from the previous occupation and serves as an anchor to the workers’ career path based on the notion that occupation mobility is often limited in the short term.^10^

Occupation recommendation algorithms should address possible general equilibrium spillovers through congestion (Naya et al., 2021). As the measured worker profiles are likely to be heterogeneous, our distance measure also proposes heterogeneous recommendations, resulting in relatively little congestion. In the two previously conducted RCTs, our distance measure most often recommends the occupation “clerical office worker” as the first occupation, which features a share as low as 4.39% (Benghalem et al., 2023).

### Role of the measured descriptors

In this section, we set $\alpha = 0$ in Eq. 4 and thereby abstract from information based on the match between an occupation and a worker’s profile. The distance measure then consists solely of information concerning occupational profiles, as measured using O*NET data.^11^ In the following, we discuss the extent to which the measured descriptors can proxy the richness of the occupation descriptors.

**Fig. 5:**
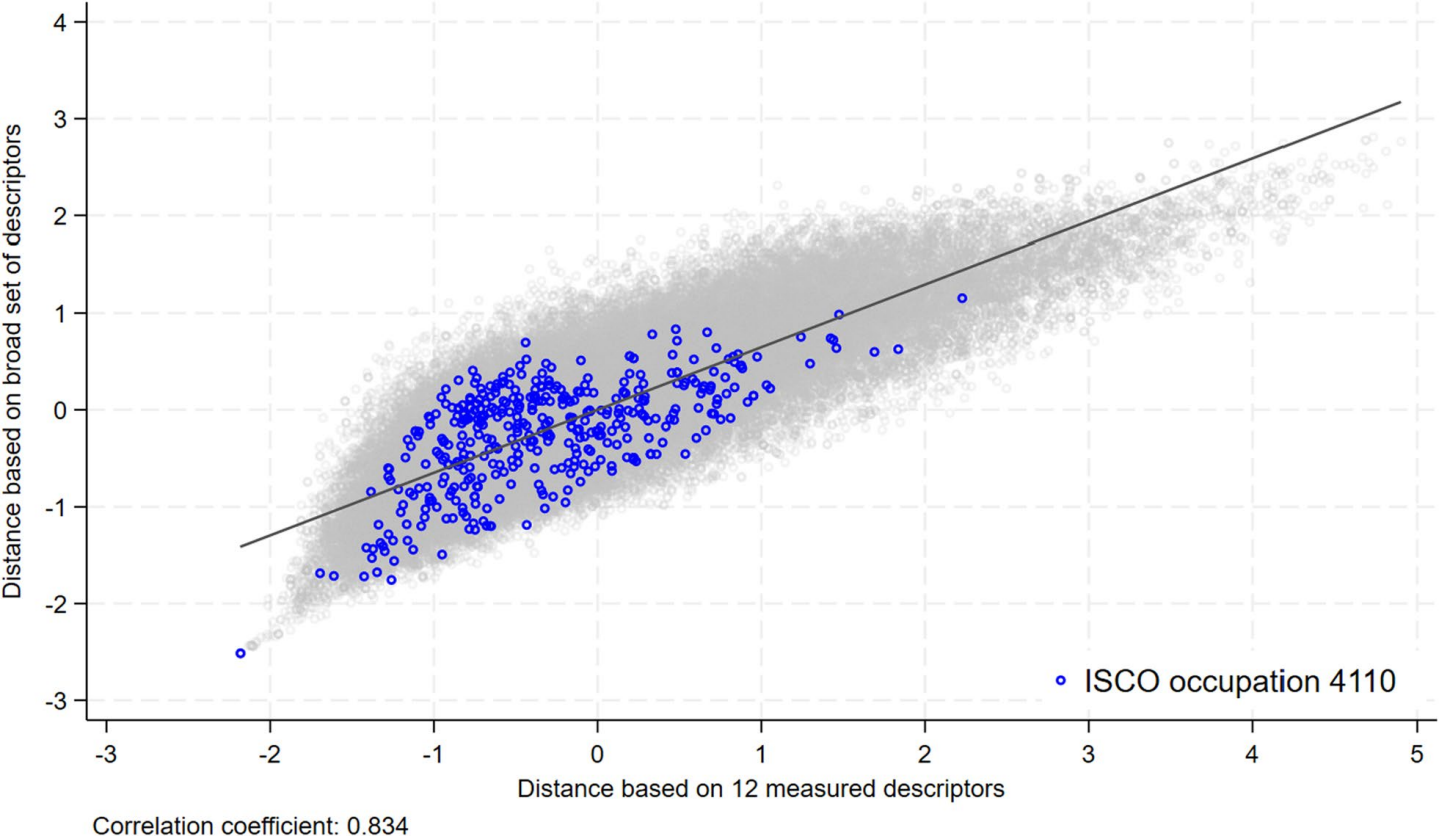
Narrow versus broad distance measure across occupations. *Note:* The x-axis shows the Euclidean distance between any occupation pair in terms of the twelve measured descriptors. The y-axis shows the Euclidean distance between any occupation pair by considering 232 other descriptors in addition. The distances between occupation 4110 and all other occupations are shown in blue. The gray dots represent the distances associated with all other occupation combinations. Source: O*NET, own calculations

In the first exercise shown in Fig. 5, we compute the distance between any occupation pair using either the measured descriptors (x-axis) or the other descriptors in addition (y-axis). We find that the distances revealed when the narrowly defined occupation profiles ($\beta =0$) are included on the x-axis explain a relatively large share of the distances revealed when the more detailed profiles ($\beta =1$) are included on the y-axis. The correlation coefficient between the two distance measures is 0.83. This pattern also holds with regard to the specific example of the ISCO occupation 4110 “clerical office worker,” which is shown in blue; this position is the most common occupation in Switzerland.^12^ Adding more descriptors tends to increase the distance associated with occupation pairs featuring a relatively short distance, which are located at the left of Fig. 5, and to decrease the distance associated with occupation pairs featuring a relatively large distance, which are located at the right of Fig. 5. Overall, increasing $\beta$ adds new information, but the distance determined by the measured descriptors already captures a substantial share of the differences among occupations.

In the second exercise, we examine the relation between the set of occupations featuring a relatively short distance and the ISCO structure. ISCO is one of the main international classification schemes, and it groups occupations based on similarities in terms of tasks and skills into ten 1-digit groups that can be further disaggregated into 4-digit groups.^13^ We are interested in the share of occupations that belong to the same ISCO group as the reference occupation (e.g., previous occupation) for both low and high $\beta$. In the following, we restrict our sample to the ten occupations that exhibit the shortest distance for each 4-digit occupation, i.e., the occupations that are likely to be recommended by a recommendation engine (Benghalem et al., 2023).^14^

**Fig. 6:**
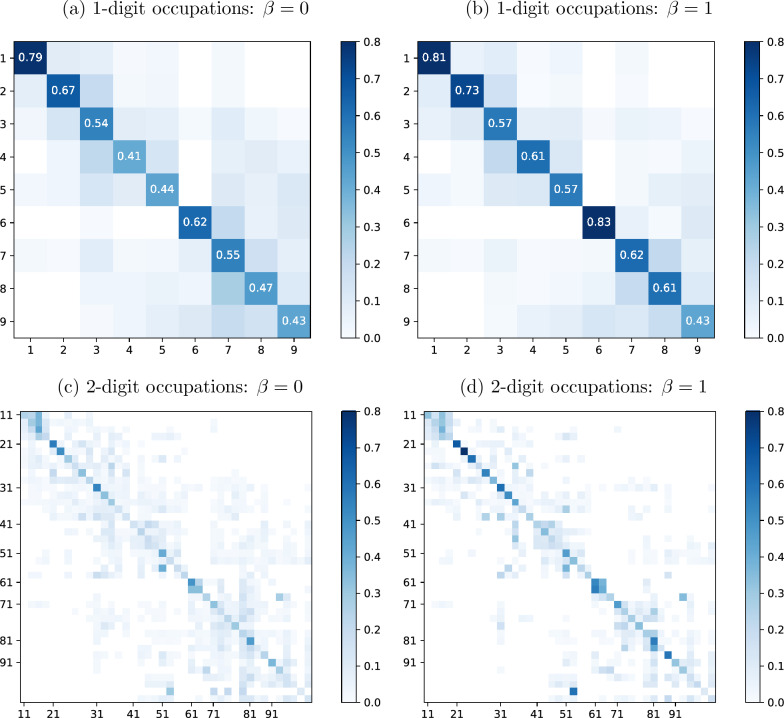
Share of recommended occupations by ISCO group. *Note:* This figure shows the shares of recommended occupations within the same 1- or 2-digit occupation group according to ISCO when $\beta =0$ and $\beta =1$, respectively. The sample of recommended occupations consists of the ten occupations that exhibit the shortest distance with regard to each 4-digit occupation. The numbers shown along the diagonal in Panels** a** and** b** represent the percentage size of occupation recommendations within the same occupation group depending on $\beta$. In Panels** c** and** d**, the first 2-digit occupation of a 1-digit group is indicated along the axes. Source: O*NET, own calculations

**Fig. 8:**
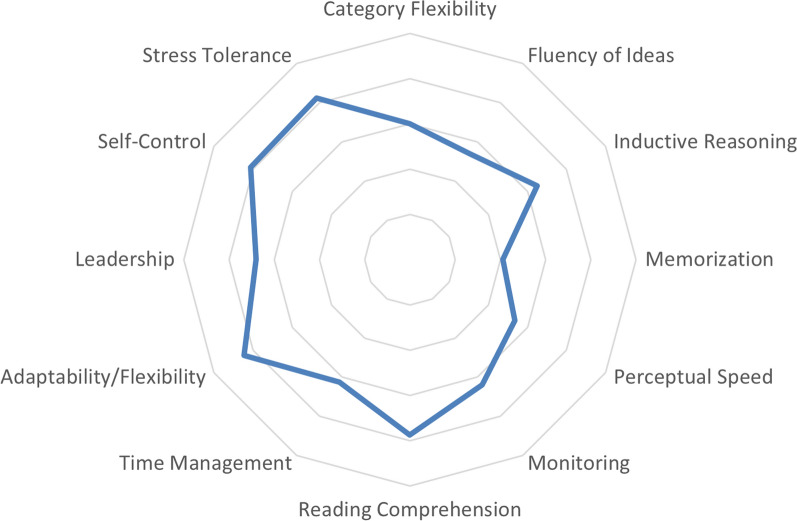
Importance of the measured descriptors for the occupation “clerical office worker.” *Note:* This figure illustrates the importance of the twelve measured descriptors with regard to the occupation “clerical office worker” (ISCO 4110). Source: O*NET, own calculations

Figure 6 presents the share of recommended occupations within a 1- or 2-digit ISCO group depending on the size of $\beta$. The ISCO group on the x-axis refers to the reference occupation $o_i$, while the ISCO group on the y-axis refers to the recommended occupation *o*. Each cell indicates the share of recommended occupations that fall within a specific ISCO group by reference occupation. When setting $\beta =0$ and thus using information drawn solely from the measured descriptors, on average, 54.7% of the occupation recommendations are from the same 1-digit ISCO group as the reference occupation, as shown along the diagonal in Fig. 6a. These shares vary across ISCO groups from a minimum of 41% for ISCO group 4 to a maximum of 79% for ISCO group 1.^15^ Increasing $\beta$ leads to more overlap with the ISCO structure, especially with regard to ISCO occupations 4 to 8 (Fig. 6b). These occupations require a “high level of manual dexterity” according to the ISCO and map to a skill level of two, thereby requiring a lower-secondary or upper-secondary education. The shares observed within the same ISCO group are considerably lower when focusing on the 2-digit classification: these figures range between 10% (ISCO groups 82, 94 and 95) and 56.3% (ISCO group 21) when $\beta =0$, as shown in Fig. 6c. A mechanical explanation of the observation of generally lower shares in this context than with respect to the 1-digit ISCO is that not every 2-digit ISCO group includes at least ten 4-digit occupations.^16^ With few exceptions (ISCO groups 12, 13, and 91), the share of recommended occupations within the same 2-digit ISCO group increases as $\beta$ increases (Fig. 6d).

Overall, the pattern shown in Fig. 6 suggests that a focus on a narrow set of individual characteristics when measuring proximity among occupations leads to a broader set of occupations that exhibit a potential fit with a worker than the set associated with the ISCO structure. When the distance measure is enriched with a variety of occupation descriptors, the recommendations are more in line with the structure of the ISCO because they are more strongly anchored on the reference occupation.

### Role of mismatch

In this section, we consider the role of $\alpha$ and set $\beta =0$ in Eq. (4). Given that $\alpha >0$, we take into account information regarding the match between a worker’s profile and the reference occupation. When no mismatch is evident (i.e., $i = o_i$), the worker’s profile is not informative and occupation recommendations are in line with the case when $\alpha =0$ (see Sect. 4.2). When a mismatch is evident, i.e., $i \ne o_i$, we are interested in how the degree of mismatch and the size of $\alpha$ change the composition of the recommended occupations.

We simulate worker profiles by adding uniformly distributed random numbers to the O*NET occupation data regarding the measured descriptors. In the following example, the simulated profile is based on the reference occupation 4110 “clerical office worker.” When no mismatch is evident or when $\alpha =0$, seven of the ten closest occupations are from the same ISCO group 4. Table 3 provides an overview of how many of these ten occupations are from the same ISCO 1-digit occupation group as the reference occupation on average when a mismatch is introduced. The number of occupations within the same ISCO group decreases as $\alpha$ increases. The greater the mismatch is, the more rapidly this number decreases. This pattern is consistent with the claim that if the previous occupation does not seem to be a good fit, our measure recommends occupations that are different from that previous occupation.

**Table 3:** Mean number of top ten occupations exhibiting the shortest distance from the same ISCO 1-digit group as reference occupation 4110

Type of mismatch	$\alpha =0.1$	$\alpha =0.3$	$\alpha =0.5$	$\alpha =0.6$	$\alpha =0.7$	$\alpha =0.9$	$\alpha =1$
Mismatch $\sim U(-0.5,0.5)$	7.970	7.589	6.520	5.963	5.338	4.374	4.101
Mismatch $\sim U(-1,1)$	7.724	6.917	5.644	5.011	4.399	3.573	3.309
Mismatch $\sim U(-2,2)$	7.446	6.364	4.638	3.721	2.995	2.155	1.910
Mismatch $\sim U(-3,3)$	7.392	6.043	3.833	2.854	2.144	1.447	1.226

This table shows the mean number of the top ten occupations that are from the same 1-digit ISCO group as the reference ISCO occupation 4110 across all simulations. Simulated numbers based on 1,000 repetitions. Source: O*NET

**Table 4:** Probability that the previous occupation is included among the top ten occupations featuring the shortest distance

Type of mismatch	$\alpha =0.1$	$\alpha =0.3$	$\alpha =0.5$	$\alpha =0.6$	$\alpha =0.7$	$\alpha =0.9$	$\alpha =1$
Mismatch $\sim U(-0.5,0.5)$	100%	100%	100%	97.5%	72.3%	17.3%	8.0%
Mismatch $\sim U(-1,1)$	100%	100%	99.5%	86.1%	57.7%	19.9%	12.0%
Mismatch $\sim U(-2,2)$	100%	100%	90.3%	66.5%	39.1%	12.3%	6.5%
Mismatch $\sim U(-3,3)$	100%	100%	81.8%	50.7%	24.4%	7.3%	4.0%

This table shows the probability that the reference ISCO occupation 4110 is part of the top ten recommended occupations across all simulations. Simulated shares based on 1,000 repetitions. Source: O*NET

Table 4 shows that the likelihood that the reference occupation is in the set of the top ten occupations that exhibit the shortest distance decreases as $\alpha$ and mismatch increase. The reference occupation remains in the set of recommendations up to an $\alpha$ of approximately 0.5 independent of the type of mismatch. This persistent link to the reference occupation is an important feature of our measure that can help job seekers understand the set of recommendations.

Overall, both tables suggest that in cases featuring mismatch (or measurement error) and $\alpha < 0.5$, the set of recommended occupations is composed of occupations that exhibit a relatively high degree of similarity to the ISCO group of the previous occupation. However, the higher the value of $\alpha$ becomes, the less the recommendations depend on the previous occupation.

### Discussion

Our approach to recommending occupations uses information concerning both the distance between a measured worker profile and the profile of any occupation, and the distance between the previous occupation profile and the profile of any occupation. In this section, we discuss how our approach to the task of measuring distances compares with other measures. We compare occupation recommendation approaches in terms of the share of recommendations that lie within the same 1-digit ISCO category as the previous occupation (referred to as “share within same ISCO group”). This indicator provides information regarding how closely recommendations are linked with the previous occupation in terms of the ISCO structure. As a benchmark, we now calculate the share of occupations within the same ISCO group for our distance measure. We abstract from the worker profile (i.e., $\alpha =0$ and $\beta =1$ in Eq. 4). We find that 63.3% of the ten occupation recommendations are within the same 1-digit ISCO occupation as the reference occupation (see Fig. 6 for shares by 1-digit ISCO category).

In a seminal paper, Gathmann and Schönberg (2010) investigate how transferable task-specific human capital is. They construct a task-specific measure based on information concerning whether a given task is performed in the context of an occupation by reference to survey data collected from German employees over four waves between 1979 and 1999. They construct a dataset including 64 occupation groups and information regarding 19 tasks that are aggregated into analytic, manual and interactive tasks. To calculate the share within the same ISCO group, we assign a 4-digit ISCO number to each occupation name, resulting in 86 different ISCO occupations. We then calculate the Euclidean distance across the three types of tasks for all occupation pairs. Most of the occupations documented in Gathmann and Schönberg (2010) belong to the ISCO categories 2 (17 occupations), 5 (10 occupations), 7 (31 occupations), or 8 (14 occupations). We use occupations from these four categories as reference occupations and identify the ten closest occupations with respect to task distance. We find that 56.1% of the ten occupations that exhibit the shortest distance are within the same 1-digit ISCO category as the reference occupation.

Belot et al. (2019) provide job seekers with online advice concerning suitable occupations by using two methodologies. The first such methodology exploits actual labor market transitions observed in the British Household Panel Survey and the national statistical database of Denmark. The authors link the preferred occupation of job seekers to the most common occupation to which people employed in the preferred occupation transition, an approach which is similar to that adopted in other papers (Schubert et al., 2020; Altmann et al., 2022; Barbanchon et al., 2023). The second methodology, which is similar to ours, shows ten occupations with similar skill requirements as the preferred occupation according to O*NET data (Allen et al., 2012). This methodology is based on 167 descriptors drawn from the subdomains of knowledge, skills, work activities, work context and job zone, which represents a subset of the descriptors that we include in the $\beta$-term in Eq. (4). We calculate the share within the same ISCO group using O*NET data, which are available with regard to 8-digit SOC occupations, in which context each reference occupation has a maximal number of ten recommended occupations as ranked by an index. We use our SOC-ISCO crosswalk and transform the 8-digit SOC occupations to 4-digit ISCO; then, for each ISCO reference occupation, we keep the ten recommended occupations featuring the lowest index numbers. We find that 45.02% of the recommended occupations are within the same 1-digit ISCO group as the reference occupation.

**Table 5:** Comparison of approaches

Distance measure	Data source	Share within same ISCO group (%)
This paper	O*NET, several domains	63
Gathmann and Schönberg (2010)	Task intensity according to German surveys	56
Belot et al. (2019)	O*NET, several domains	45

This table shows how the distance measure we use to recommend occupations compares to other measures. The variable “Share within same ISCO Group” refers to the share of the ten closest occupations that are within the same 1-digit ISCO group as the reference occupation

As summarized in Table 5, the approach developed by Belot et al. (2019), which relies on O*NET’s occupation recommendations, provides the broadest suggestions; namely, in this context, only 45% of the suggestions are within the same ISCO group. Gathmann and Schönberg (2010), who construct a task distance measure, provide recommendations that are almost equally likely to be in the same ISCO group. Our approach tends to provide recommendations that are more likely to be within the same 1-digit ISCO group than these two alternative approaches. When we abstract from the measured worker profile (i.e., $\alpha =0$), the first recommended occupation is always the same as the reference occupation, thus artificially increasing the number of occupations within the same ISCO group. Overall, all three approaches provide occupation recommendations from the same 1-digit ISCO group, which are likely to be similar to the previous occupation, as well as from other ISCO groups, which are likely to contain more novel information.

## Conclusion

Information friction can prevent workers from finding a suitable job. This issue is likely to be exacerbated in periods featuring increasing technological change, as certain occupations may disappear and new occupations may emerge. To overcome such frictions, we introduce a Euclidean distance measure that proposes worker-specific occupation recommendations based on the proximity between those occupations and a worker’s profile. This measure takes into account information drawn from a worker’s profile that is related to skills, abilities and work styles as well as the previous occupation. We identify a set of relevant worker characteristics and design an online assessment to measure the worker’s profile. By combining this profile with O*NET data concerning occupational profiles, our measure allows occupations to be ranked in terms of how well they match a given worker.

The worker’s profile can deviate from the profile of past occupations and does not take educational requirements into account. This approach may result in recommendations that are not feasible in the short term due to a lack of education or situations in which employers require specific work experience. While these mobility restrictions are present in the labor market, our distance measure can be specified in a flexible manner by including parameters that assign different weights to the measured characteristics that capture the worker’s profile as well as to a broad set of other characteristics, which can be used to capture work experience.

Our newly developed distance measure can support job seekers by providing them with information regarding their mismatches with a variety of occupations. Such information is especially helpful for workers who have been displaced from their previous jobs, for example, due to structural change or low productivity. Our tool for generating worker-specific occupation recommendations is cost-effective and scalable; accordingly, it can be used by policy makers, public institutions and businesses.

## Figures and Tables

**Fig. 7:**
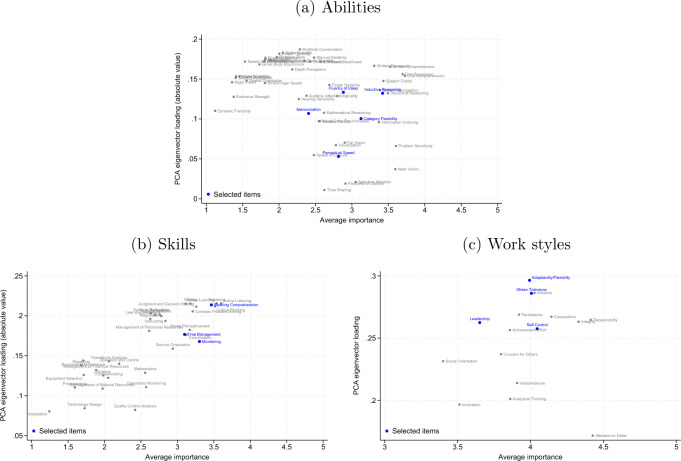
Importance and information content of descriptors by subdomain. *Note:* The descriptors selected to measure a worker’s cognitive profile are shown in blue. All other descriptors within a specific subdomain are shown in gray. The first principal component eigenvector of the abilities subdomain explains 46.18% of the variation, while those of the skills and work styles subdomains explain 46.95% and 51.97%, respectively. Source: O*NET, own calculations

## Data Availability

Our main data source is O*NET, which is publicly available here. We also rely on data from Benghalem et al. (2023) in Subsect 3.4. These data will be made available after the publication of the corresponding manuscript. The study Benghalem et al. (2023) is approved by the Ethical Committee of the HEC University of Lausanne.

## Appendix

See Figs. 7, 8.

## Abbreviations

ISCO: International Standard Classification of Occupations
O*NET: Occupational Information Network
PCA: Principal component analysis
RCT: Randomized controlled trial
SOC: Standard Occupational Classification
VCV: Variance–covariance

## Footnotes

1 For instance, job seekers who have not yet accumulated work experience in a previous occupation may want to receive recommendations that are based solely on their measured profile. Conversely, job seekers who would like to remain in their previous occupation or in a closely related occupation may assign a high weight to the requirements of their previous occupations.

2 Specifically, condition a) requires that $\bar{w_{j}}=\sum _o^O w_{oj}/O$ is larger than a threshold. With regard to condition b), we identify descriptors using principal component analysis. Specifically, let $w_j$ be a column vector that contains information concerning descriptor *j* for all occupations. Let $W=(w_1,...,w_J)$ be a (mean centered) matrix of occupation requirements. The variance–covariance (VCV) of the importance values for all occupations is $W'W$. The principal component decomposition of the VCV is $W'W = E \Lambda E'$, where *E* is the matrix of eigenvectors and $\Lambda$ is the diagonal matrix with eigenvalues. We select the first principal component; thus, $W'W\approx E_1 \lambda _1 E_1'$, where $E_1$ is the first principal eigenvector, and $\lambda _1$ is the largest eigenvalue. $W \approx E_1 \sqrt{\lambda }$. $W'E_1$ is the matrix featuring nonnormalized loadings. We select cases featuring high loadings.

3 O*NET is designed to provide an accurate representation of the US occupational structure, but its use in the context of other industrialized countries has been validated by a number of studies, including in the case of Switzerland (Forstmeier & Maercker, 2008; Hanna et al., 2019).

4 O*NET asks “How important is *[input name of descriptor]* to the performance of your current job?”; targets are asked to respond to this question on a scale ranging from 1 (not important) to 5 (extremely important), and the answers are used to determine the importance of a descriptor. To measure the level of a descriptor, the following question asked is asked: “What level of *[input name of descriptor]* is needed to perform your current job?” Targets are asked to respond to this item on a scale ranging from 1 to 7, with descriptor-specific examples provided at levels 2, 4 and 6. For example, O*NET provides the following references for the descriptor Reading Comprehension (skill): level 2, “Read step-by-step instructions for completing a form,” level 4, “Understanding an email from management describing new personnel policies,” and level 6, “Read a scientific journal article describing surgical procedures.”

5 Note that we also account for descriptors that exhibit high loadings in the second principal component that explains 16.37% of the total variation in the importance value.

6 We use data drawn from two randomized controlled trials (RCT) that were conducted in the Swiss cantons of Neuchâtel and Vaud between 2021 and 2023. All registered job seekers between the ages of 18 and 65 years were eligible to participate. The Public Employment Services invited registered job seekers by sending emails that contained treatment-specific registration URLs, which were randomly allocated. Completing the online assessment was a precondition for participation in the study. The total number of participants was 1,547.

7 To define the occupation profiles, we use data drawn from O*NET. We take the value of the level scale for skills and abilities to be consistent with the data collected through the online assessment and the value of the importance scale pertaining to work styles because these data represent the only available information in this context.

8 We specifically consider the other worker-oriented descriptors that we ignore when measuring the worker profile as well as descriptors from the job-oriented but cross-occupation category occupational requirements. These descriptors, which are drawn from nine subdomains, represent the set of descriptors that O*NET uses in the related occupations matrix (Allen et al., 2012) and are as follows: all subdomains from the worker characteristics domain (abilities, interests, work values, and work styles); the subdomains skills and knowledge from the worker requirements domain; the subdomains work activities and work context from the occupational requirements domain; and information regarding the Job Zones that group occupations into five categories based on the levels of education, experience and training they require. With regard to work styles, we use the importance scale, as it is the only scale available in this context. With respect to abilities, skills, knowledge and work activities, we use the average value of the two available scales, i.e., importance and level. Regarding the work context, we use the scale context (CX); concerning interests, we use the scale interest (OI); and with regard to work values, we use the scale value (EX).

9 Each of the eleven Euclidean distance matrices is standardized (*z*-score) because descriptors may be associated with different scales across subdomains, thus affecting the range of distances.

10 The distance measure included in Eq. 4 has been used in two experiments. In an RCT conducted in the French-speaking area of Switzerland, the participants could choose the magnitudes of $\alpha$ and $\beta$, thus offering them a high degree of flexibility with regard to setting the focus of the occupation recommendations (Benghalem et al., 2023). In an ongoing RCT that focuses on the German-speaking area of Switzerland, the parameters are specified in advance by the research team for different treatment arms.

11 We first transform the SOC occupation classification into the 4-digit ISCO-08 using two crosswalks. The first crosswalk transforms the 8-digit SOC19 into the 8-digit SOC10. The second crosswalk transforms the 6-digit SOC10 into the 4-digit ISCO-08. O*NET provides information regarding 923 occupations in terms of the 8-digit SOC19. With complete information on all necessary subdomains following Eq. 4, 873 8-digit and 759 6-digit SOC occupations remain. The application of the crosswalk to the O*NET data results in 422 4-digit ISCO-08 occupations. We use the average when collapsing the data.

12 According to the Swiss Federal Statistical Office, between 2019 and 2021 an average of 5.3% of the employees in Switzerland worked in this occupation. This value represents the highest share across all 4-digit occupations.

13 The ten 1-digit occupation groups are as follows: 1. manager, senior officials and legislators; 2. professionals; 3. technicians and associate professionals; 4. clerical support workers; 5. services and sales workers; 6. skilled agricultural and fishery workers; 7. craft and related trades workers; 8. plant and machine operators, and assemblers; 9. elementary occupations; and 0. military occupations. See also https://www.ilo.org/public/english/bureau/stat/isco/isco08/.

14 Note that by construction, the previous occupation is the occupation that exhibits the shortest distance because their profiles coincide, i.e., $b_{o_i} = b_o$. Varying $\beta$ thus affects the ranking of occupations beyond the occupation that exhibits the shortest distance.

15 Note that these shares do not account for different numbers of occupations within 1-digit groups. These numbers range from 18 4-digit occupations for the 1-digit group “6” to 92 4-digit occupations for the 1-digit group “2.” The correlation between the share of recommended occupations shown in Fig. 6a and the number of 4-digit occupations within a 1-digit group is 0.13 and not significant.

16 ISCO group 82, which features three 4-digit occupations, and group 95, which features two occupations, exhibit the lowest number of occupations. The ISCO groups 21 and 33, both with 24 occupations, and group 81 with 26 occupations have the highest number of occupations.
